# Anti-corruption, Transparency and Accountability: Case Study of Healthcare in the Arab Countries

**DOI:** 10.1080/16549716.2019.1704529

**Published:** 2020-03-20

**Authors:** Mostafa Hunter, Rania Uwaydah Mardini, Arkan El-Seblani, Sammer Elsayed

**Affiliations:** a Senior Independent Anti-Corruption Consultant; b Olayan School of Business, American University of Beirut, Beirut, Lebanon; c Anti-Corruption, UNDP, Arab States; d Independent Health Governance Consultant

**Keywords:** Anti-Corruption, Transparency and Accountability, Corruption, risk, governance, health

## Abstract

**Background**: The Arab states suffer from high levels of corruption. The UNDP’s team there developed an approach to tackle corruption and enhance transparency and accountability in healthcare as part of its broader efforts to support the Sustainable Development Goals. This work evolved into a proper tool, the Conceptual Framework for Corruption Risk Assessment at Sectoral Level (hereafter ‘Framework’), with implementation guides that enable tailoring to sector and country context.

**Objectives**: This article documents the development of the Framework, its methodology and observed added value.

**Methods**: Qualitative methods were utilized comprising desk research, field experience, stakeholder outreach, and focus group observation and documentation. It was most appropriate because the objective was to develop a methodology with specific characteristics.

**Results**: The new approach uses anti-corruption as an explicit entry point to governance reforms. It articulates a structured evidence-based method to apply risk management methodology – tailored to the specificities of corruption as a risk – in healthcare whereby assessment and mitigation are (a) within institutions (b) focused on decision points and (c) around transactions while bringing together health and anti-corruption communities towards designing measurable results-oriented reforms.

**Conclusions**: The Framework may be effective in driving concrete governance reform efforts that demonstrably reduce corruption by means of creating a common language and agenda among different stakeholders, changing the mindset towards reform, and developing targeted solutions with higher return on investment. As such, it may be capable of generating observable and sustainable progress towards healthcare reform.

## Background

Sound health is fundamental to sustainable economic and social development. Thus, historically, massive efforts and funds have been invested in this critical sector with significant results; yet, they fall short of desired objectives. For instance, at least 50% of the global population remains deprived of fundamental health services, with all the repercussions that this entails including, increased poverty levels and diminished quality of life [1].

The challenges to increasing access to essential health services are numerous and continuously evolving due to environmental, economic, political, and social factors and the interplay amongst them. One such major challenge is corruption [2,3]. Corruption, defined by Transparency International as the ‘the abuse of entrusted power for private gain’, creates an environment where health budgets are misappropriated, health-care providers demand bribes, and fake or contaminated medication infiltrates markets [4]. In turn, people are deprived of proper access to medical facilities, supplies and staff, health challenges are inadequately addressed, and policy implementation is undermined. Besides the millions of health-care dollars lost to corruption annually, the quality of life is significantly compromised [2]. In the worst cases, human lives are actually lost [5,6].

Against this backdrop, healthcare is one of the core themes of the 17 Sustainable Development Goals (SDGs) adopted by world leaders in 2015; it is the focus of the third SDG, concerns at least 10 others; and relates to over 50 SDG indicators [1]. *Universal health coverage*, a term referring to essential health services being of sufficient quality, accessible to, and affordable by everyone, is *a key objective of SDG 3 and* a top priority for the World Health Organization (WHO) [1]. Good governance, which encompasses the ‘wide range of […] functions carried out by governments/decisions makers as they seek to achieve national health policy objectives […]’ [1], is another key theme of the 2030 global development agenda that emerges in SDG 16 on ‘Peace, Justice and Strong Institutions’. It is the underpinning of any strong, effective ecosystem such as the health sector, for instance [7]. Corruption massively undermines the effectiveness of any given structure; it has therefore been a central target of governance reforms [8]. However, targeting governance reforms as an entry point is, in effect, a circular logic approach to the problem: governance reforms should address corruption, but corruption could undermine any attempts at such reforms to begin with. The United Nations Development Program (UNDP) wanted to test an alternative hypothesis which could avoid this problem: targeting corruption should be the starting point for other governance reforms and enhanced transparency and accountability. From this effort emerged a new tool.

## Objectives

The objective of this paper is to document the development of the aforementioned new tool, its methodology and observed added value.

### UNDP’s anti-corruption program in the Arab countries: an overview

UNDP’s efforts to address corruption in the Arab region are primarily carried out by its Anti-Corruption and Integrity in the Arab Countries (ACIAC) program [9]. By the end of its first operational phase (2011–2014), UNDP-ACIAC had supported the establishment of multi-stakeholder networks and processes for the implementation of the United Nations Convention Against Corruption (UNCAC) and has helped several countries develop legislative frameworks and technical capacities in this regard. By the start of its second phase (2015–2018), ACIAC is building on previous work while expanding it to targeted initiatives in key vulnerable sectors, including health and customs, while empowering new actors to engage in collective action including civil society, the business community, and youth groups. Over the years, UNDP-ACIAC has come to identify key trends in national anti-corruption strategies and gaps that undermine their success; and has developed various methods and approaches to help national stakeholders to address those gaps, often drawing on the added value of the regional platform it has created and cemented over the years.

### An innovative approach to anti-corruption

National anti-corruption strategies [10–12] to date have mostly targeted general overarching issues such as enhancing legal frameworks, setting up anti-corruption agencies, promoting investigation and ensuing enforcement; and/or raising awareness as to the corrosive effects of corruption. Notwithstanding the value of these strategies in promoting an environment that is generally conducive to integrity, their effectiveness and resource-efficiency is suboptimal as they are either too broad (rather than targeted), or they are reactive (rather than preemptive), and they are indifferent to contextual differences. There is a need for additional complementary strategies that are pragmatic and targeted, pre-emptive, and context-sensitive. With this objective in mind, UNDP-ACIAC adopted a different approach that it would soon develop into a novel tool, the Conceptual Framework for Corruption Risk Assessment at Sectoral Level (hereafter ‘Conceptual Framework’ or ‘Framework’).

## Methods

The methods used to formulate the risk assessment approach were qualitative in nature and comprise mainly desktop research, review of field experience, stakeholder outreach, and focus group observation and documentation. It is most appropriate, first, because the objective is to develop a methodology. Second and as aforementioned, the success of this methodology requires the buy-in and engagement of multiple stakeholders; therefore, the collection of inputs from these stakeholder groups and the observation of the dynamics between them are necessary to inform the formulation process which comprised two phases as follows:

### Phase I: initial development and conceptualization of the framework (October 2015 – September 2016)

The first phase of the project reflected on existing anti-corruption efforts and on how these may be customized to specific sectors, namely health, so as to be better targeted, more effective, and in synergy with the newly adopted 2030 Global Agenda for Sustainable Development. The following methods were mainly used:

- Comprehensive desk research.
- One-to-one semi-structured interviews with relevant key informants﻿.
- Two regional expert group meetings/focus group discussions.

#### Selection of the participants

Selection of the key informants for the one-to-one interviews aimed at achieving diversity to ensure the maximum variability within the data collected and to allow comparability of the different perspectives. The participants were targeted in a way that ensures representation of different types of relevant stakeholders (public, private for profit, private not-for profit, civil society, academia, subject matter experts and international consultants and representatives of multilateral organizations) with regional and global experiences. A total number of seven key informants were approached directly by the researcher through an email or via phone. The background aims of the project were clearly explained to all participants either verbally by the researcher of through an information sheet attached in an email.

For the two regional expert groups meetings, a total of 36 participants with specialized knowledge relevant to the health sector and the anti-corruption realms were selected. They were approached by email and invited to attend a 2–3 day workshop, the background, objectives, and expected outcomes of which were outlined clearly.

#### Data collection

Comprehensive desk research comprised mainly review of existing literature on anti-corruption efforts.

In the semi-structured interviews, the participants were asked about topics related to processed relevant to their areas of work, corruption and potential corrupt acts as per their perception and the components of the health sector functions, areas and the underlying decision points, potential deviation in each decision point and the impact of each deviated decision. The average duration of each interview was 60 min. The interviews were conducted either personally, online or via telephone.

In the regional expert group workshops, the participants were first introduced to the main functions of the health sector and general concepts of anti-corruption efforts because their backgrounds typically related to either health or anti-corruption, but not both so there was a need to bridge the gaps. The participants were stimulated to undertake interactive discussions. In addition, the participants were divided into six working groups each included around 4–5 participants where they were asked to perform an initial assessment of risks to corruption within the health sector area specific to the group. A rapporteur was appointed to record the feedback of the discussions and the working group activities.

The first key outcome was the conceptualization of the risk assessment methodology including:

- Main elements and the interrelation between them: deviated decisions relating to decision points, identified within decision areas, mapped to domains, within sector functions within sectorial objectives, (see Figure 2)
- The process of assessing likelihood as a function of drivers and restraints,
- The process of assessing impact, as a function of the total number of sectoral objectives affected and the dimension of the effects.

Another main group of findings pertains to health sector specificities essential for conducting the corruption risk assessment: the five health sector functions (see Figure 3) and typical underlying decision points, potential deviations in each and the areas of impact of such deviation.

### Phase II: pilot implementation of the proposed framework in selected countries and territories (September 2016 to January 2018)

The Conceptual Framework is being piloted in six Middle East North Africa (MENA) countries and territories. A joint task force/expert group has already been formed in each, and the risk assessments are already underway. The objective is to further validate the proposed approach and to initiate the risk assessments. This phase involved the following:

- Expert group discussions
- One-to-one semi-structured interviews with the identified key informants.

It is noteworthy that the 15 months from September 2016 to January 2018 comprise the pilot phase that has further informed the Framework and this paper, but the implementation in these countries and territories is still underway.

#### Selection of the participants

Participants for the expert group discussions were selected to achieve maximum variability and ensure representation from all health sector functions in addition to stakeholders representing anti-corruption bodies. For example, the participants included representatives from health policy-making bodies, regulatory oversight bodies, health finance and insurance bodies, the pharmaceutical sector, procurement agencies and health service providers, in addition to the anti-corruption agencies. There was a total of 120 participants representing the six countries and territories with an average of 20 participants per group in each country or territory.

Key informants for one-to-one interviews were identified either from the expert group discussions or elsewhere through purposeful sampling where the intention was to get in-depth insights into the relevant issues and to ensure comprehensive representation of all the functions within the health sector in addition to the relevant anti-corruption stakeholders within each country or territory. A total of 90 participants were interviewed in the aforementioned six countries and territories with an average of 15 participants per country or territory.

#### Data collection

Eighteen focus group sessions were observed in six MENA countries and territories. Data were collected [1] to further inform the methodology of the Framework with regard to contextualization within countries and territories and [2] on the specificities of the health sector pertinent to each country and territory.

The semi-structured one-to-one interviews, with an average duration of 50–60 min, focused on corruption risks relevant to respondents’ area of work, obtained further details to feed into the initial country or territory assessment, and allowed participants to express their detailed insights and communicate their way of perceiving and assessing corruption risks within the scope of their occupation. An appointed rapporteur recorded the feedback.

It is noteworthy that the overall percentage breakdown of interviewees by function is as follows:

- 2(28.5%) Senior hospital managers (one public hospital and one private hospital).
- 1(14.2%) Financial manager of private hospital.
- 1(14.2%) Cost analytics specialist (public hospital).
- 1(14.2%) Insurance broker.
- 1(14.2%) Senior Ministry of Health official
- 1(14.2%) Fraud, audit and anti-corruption expert.

## Results

### The conceptual framework for corruption risk assessment at sectoral level [9]

The Conceptual Framework responds to the observed gaps, drawing on UNDP-ACIAC’s experience in anti-corruption efforts and research covering six countries and territories, and spanning over 2 years. These activities included two regional expert group meetings, 18 national focus group discussions, and around 100 interviews with key informants.

#### Key features of the corruption risk assessment methodology

The Framework embodies three main features. First and foremost, it integrates a focus on prevention. Prevention is grounded in risk assessment/management theory [13–15], and in contrast to enforcement which targets a few large corruption incidents after the losses have taken their toll, it aims to put controls in place that pre-empt breaches, and their related losses altogether, and/or detect them in a timely manner [13]. Moreover, the detective controls enhance the effectiveness of criminalization and enforcement measures the deterring effect of which is closely correlated with the likelihood of detection [16]. As such, prevention is more cost-effective and enhances the returns on investments in anti-corruption efforts [13,14].

A further valuable sub-feature of prevention is the forward-looking perspective and thus the avoidance of finger-pointing. It addresses the risk that corruption could take place, related to weaknesses or opportunities in institutional systems. This approach is more likely to engender cooperation and buy-in of the different stakeholders and thus enjoys better chances of success [17].

Second, the Framework adapts to the anti-corruption realm by adopting a focus on decision points as the unit of analysis [18] [19]. From this latter emphasis derives another key feature of the Framework: its pragmatism, because attending to actual key decision points implies addressing specific-targeted vulnerabilities.

Finally, while the environmental scan and the ensuing contextual adaptation are key components of risk assessment methodology in general, the Framework goes beyond the organizational landscape to consider the specificities of the health sector within a given country context.

#### Description of the corruption risk assessment methodology

**Figure 1.**
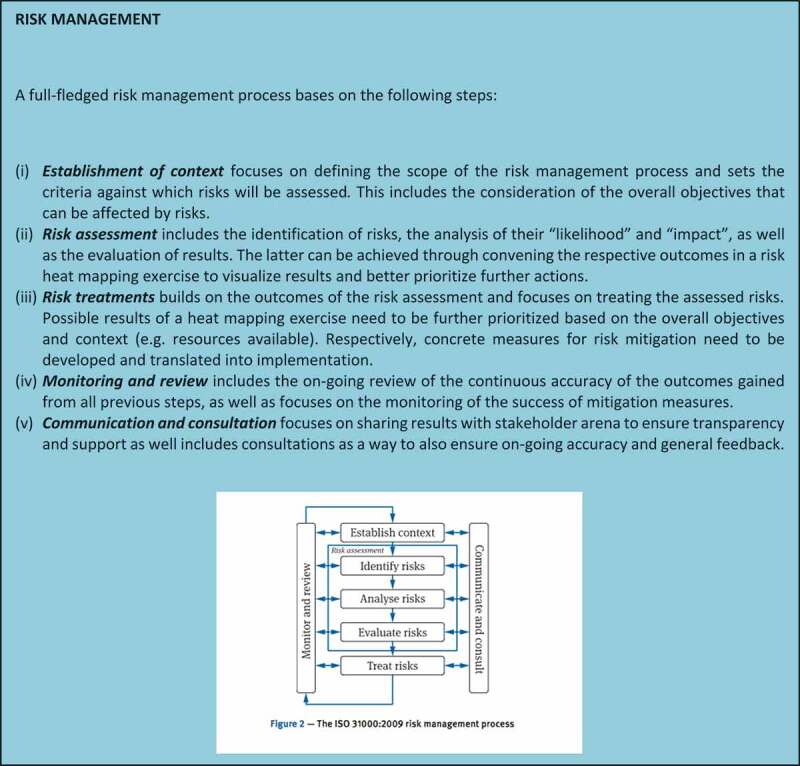
The ISO 31000:2009 risk management process.

As aforementioned, the Conceptual Framework builds on existing risk assessment/management theory [described in Figure 1] while adapting it to corruption and to the idiosyncrasies of the sector. From this, customization derives its innovation and contribution to the literature [20,21].

Core to the concept of risk management and prevention is the identification of what could go wrong in advance. To prioritize among those infinite possibilities, it becomes necessary to assess risk. Risk combines the probability of an event and its consequences [15], in other words, ‘likelihood’ and ‘impact’. Since corruption involves using one’s entrusted authority *against* the public interest as opposed to *for* it, corruption occurs during *decision-making*. The objective of this tool is therefore to identify the key decision points and assess the likelihood and impact of corruption taking place at those points. The expanded focus on decision points as a unit of analysis is a further contribution to existing literature on risk management methodology.

##### Deviated decisions

**Figure 2.**
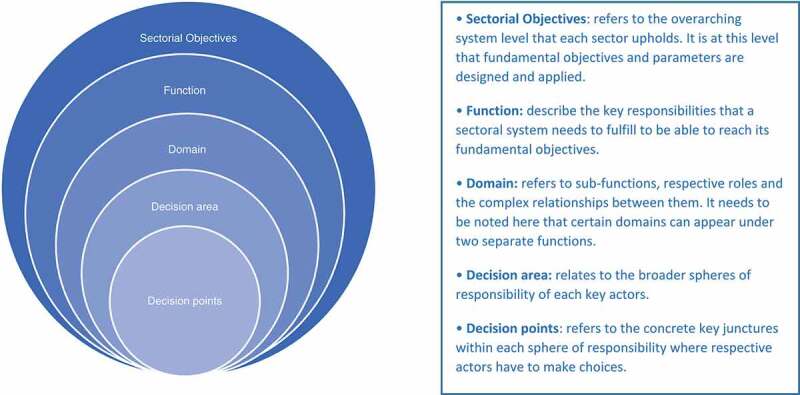
Sectoral breakdown. (Excerpted from the Conceptual Framework for Corruption Risk Assessment, UNDP-ACIAC)

The first step is to identify the decision points where corrupt activity might take place, in which case a ‘deviated decision’ occurs. Deviated decisions are defined as decisions that deviate from the targeted result by a breach of the decision maker’s duties of trust and care for private gain. Decision points are identified within decision areas mapped to the sector functions and objectives as depicted in Figure 2. All these elements (decision points, deviated decisions, decision areas, sector functions, and sector objectives) are key concepts of the Framework that were informed and developed by the research methods, particularly phase I.

**Figure 3.**
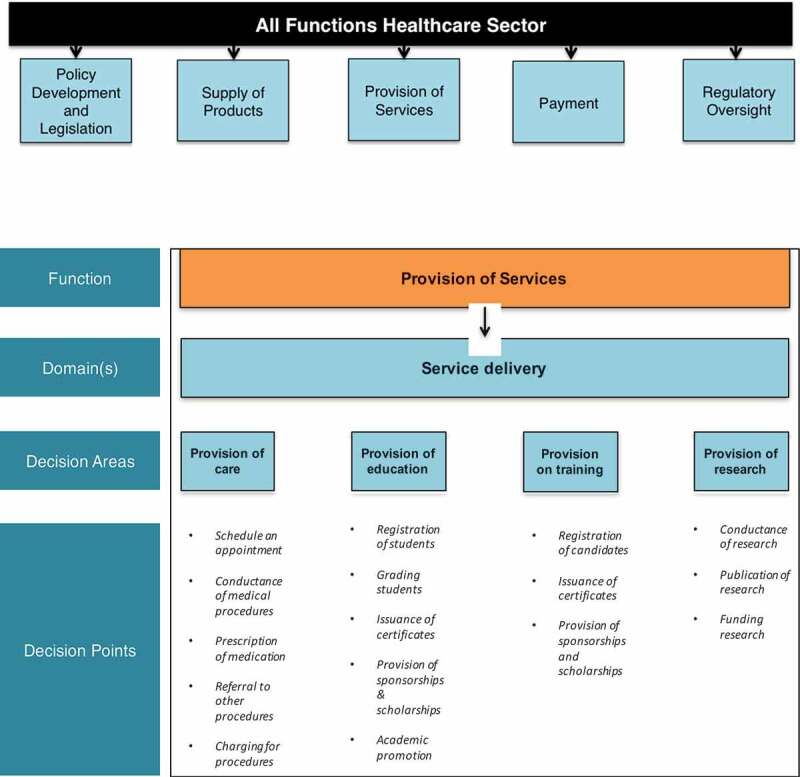
Healthcare sector functions with detail on provision of services function.

Notwithstanding that defining deviance may not always be obvious, mapping of relationships helps to bring together the different decision points, and highlights to a large extent the location of the deviated decisions. This can help stakeholders see the specific functions most affected, and the impact that deviated decisions are having on health sector objectives. The focus of this Framework is on the following functions: policy development and legislation, supply of products, provision of services, payment, and regulatory oversight. Figure 3 depicts these functions as well as the analysis of one of them, the provision of services, all the way through the decision points.

**Figure 4.**
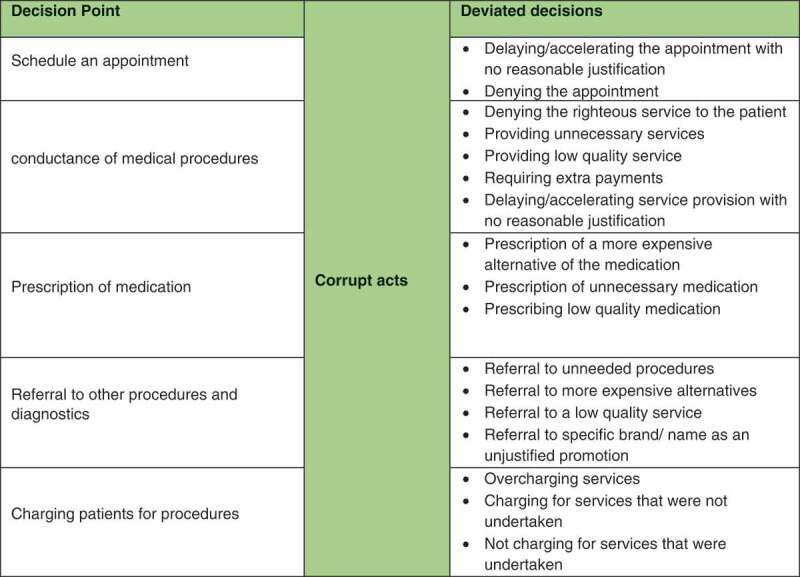
Deviated decision areas in ‘Provision of Care’ function.

Subsequently, deviated are identified at each decision point to understand how it may be affected by corrupt behavior. Figure 4 shows a sample of deviated decisions that may be associated with the ‘provision of care’ decision area:

##### Assessment of ‘likelihood’

Assessment of ‘likelihood’ is the assessment of the possibility that these deviated decisions will occur. Likelihood is a function of ‘drivers' which drive the decision-maker towards corruption. They constitute conducive factors that relate to social, political, and financial or economic pressures and/or to the nature of the transactions, related procedures, and applicable regulations. The following are a few examples of drivers:

- A polarized political situation where there is pressure to serve the political interest of one’s own group or undermine that of the opponent.
- Low remuneration where the decision-maker perceives need for a boost in income.
- Lengthy procedures that create a propensity to circumvent the red tape.
- Ambiguous procedures which create room for interpretation of the requirements and thus discretion in applying the procedures.
- Sizeable transaction value that increases the potential benefit from corrupt activity.
- The presence of informal institutions and related practices and norms that are inherently more difficult to identify and control.

‘Restraints’, on the other hand, hold the decision-maker back. Examples include:

- An anti-corruption policy.
- Ethical leadership/ ‘tone-at-the-top’.
- Strong governance systems and related internal controls that reduce the likelihood that corrupt activity will go undetected coupled with active enforcement of anti-corruption legislation.
- Declaration of assets and interests.
- Policies and procedures to prevent and manage conflicts of interest.
- Whistle-blowing protection and mechanisms: which encourage more people to expose corruption.
- A functioning legal system so that if people blow the whistle, there is likelihood of investigation, prosecution, and conviction. The size of the penalties should be relative to the context and material enough to deter corrupt behavior.
- Effective auditing.
- Heightened external scrutiny owing to the presence of watch-dogs (including civil society organizations) and advocacy for appropriate health delivery, cost, and efficiency.

##### Assessment of impact

Impact is a function of the magnitude of the distortion. It is a function of two factors [1]. The total number of sectoral objectives affected, in light of the national agenda. For example, an acquisition of fake oncological medication not only results in the waste of public funds (financial objectives) but adversely impacts the patients’ treatment (health-care objectives) [2]. The scope or dimension of the effects. For instance, and with reference to the prior example, if the volume of such purchases is large and/or there is a significant increase in mortality rates as a result then the effects are sizeable. Such a risk would thus be assessed as ‘very high impact’.

##### The risk heat map

**Figure 5.**
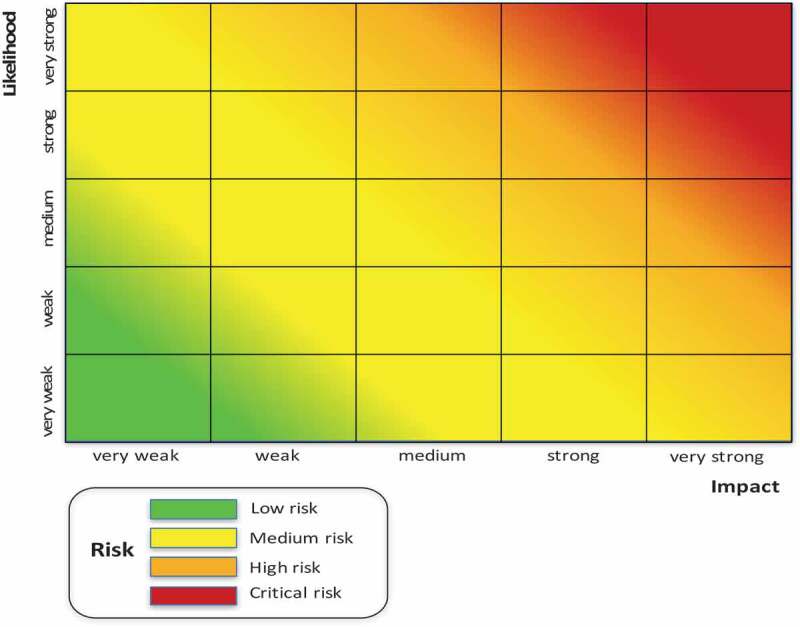
The risk heat map.

Based on the assessments of likelihood and impact, a ‘risk heat map’ (see Figure 5) is developed where the different decision points are plotted and accordingly prioritized for mitigation.

#### Case in point: registration to receive a medical service

**Figure 6.**
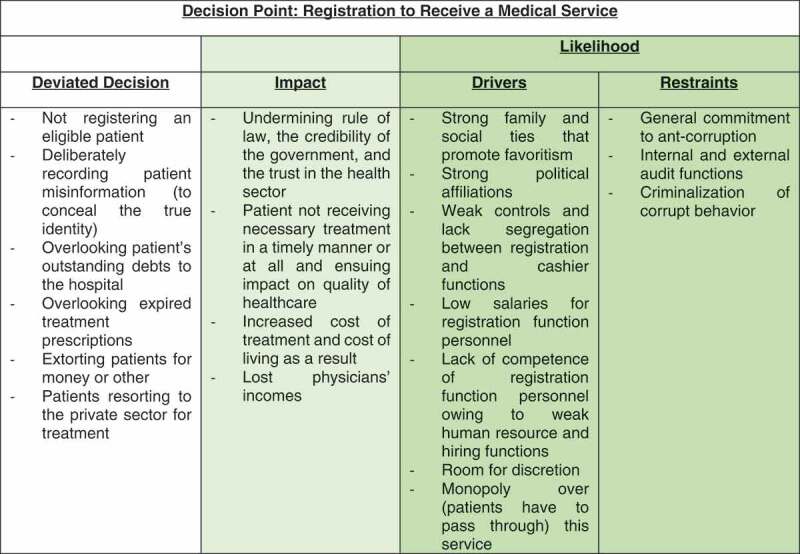
Risk assessment example.

**Figure 7.**
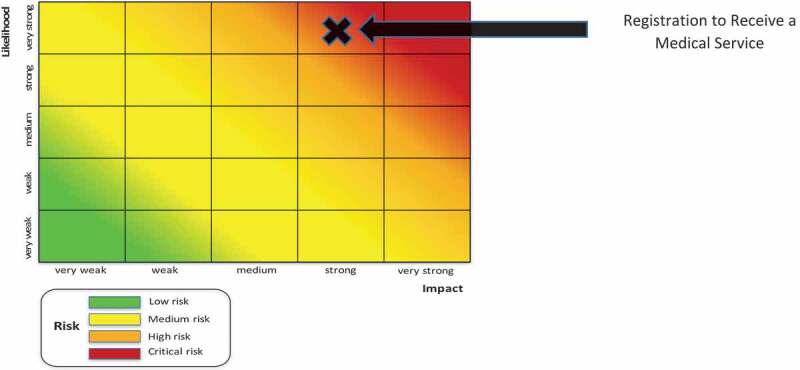
Risk heat mapping example.

Figure 6 provides an example of an actual risk assessment carried out in one of the pilot countries where the risk related to patient registration was assessed as ‘high impact and very high likelihood’. Figure 7 depicts the plotting of this decision point on the risk heat map.

#### Implementation: who? and how?

Country involvement is core to the execution of the risk assessments. Risk assessment theory requires an adaptation to and therefore an in-depth understanding of the hosting environment, including culture, practices and regulations which are typically dynamic and variable across countries and sectors. National joint task forces should be carefully formulated of diverse national stakeholders. The ‘right stakeholders’ are the various interested parties who see the issue from different angles such that together they bring a solid collective understanding of the intricacies of the sector within the local context, its corruption vulnerabilities, and from different perspectives. Ideally, the task forces would include state actors and non-state representatives (namely civil society, and private sector). The role of outside consultants or international experts, if any, should be limited to an advisory capacity. As far as actual implementation is concerned, the task forces have involved mainly the former: different departments from the Ministry of Health, anti-corruption agencies (ACAs), and government-funding bodies. Attempts to engage civil society have so far succeeded in one country; private sector, not yet.

Capacity-building activities for the stakeholders, particularly the task force members are necessary and should be designed to accomplish the following objectives:

- To convene the different stakeholders, commence a dialogue, generate a common language for and a shared understanding and ownership of the issues, and perhaps most importantly, begin to build trust. The significance of accomplishing this cannot be overemphasized basically because, given the heterogeneity of the joint task forces, there is very likely to exist prevalent lack of trust from all sides. The public sector is typically seen as the culprit because it enjoys all the power. The private sector is viewed as a significant offender given its profit-seeking nature and its propensity to therefore cut corners costs. Civil society is generally perceived as ineffective, and ACAs are understood to have a ‘policing’ role. In this environment, there is a general scapegoating of one another and lack of acknowledgement of responsibility by all parties. These issues are fueled by attitudes of distrust which may be promoted in countries that do not have experience with public-private dialogue. Add to this, the ensuing absence of a common understanding of the issues. For example, ACAs understand corruption but have little comprehension of the sector specificities; and health-care specialists are unlikely to be well versed in anti-corruption efforts and requirements.
- To develop the relevant in-house knowledge and skill that are fundamental to the sustainability of the risk assessment and management processes which, owing to the inherently dynamic nature of risk, should be ongoing.

### Some noteworthy observations

Successful anti-corruption efforts require the direct engagement of key stakeholders, and since the health sector has its idiosyncratic stakeholder environment, adopting a sectoral approach enhanced the focus on involving the right stakeholders with the greatest levels of interest, knowledge and/or influence. This enhanced participation and enriched the level of engagement and the results thereof – provided a shared understanding of overarching objectives was established at the outset. That being said, due to the high variation of the participants of the taskforce, knowledge gaps were an impediment towards understanding each other’s viewpoints and reaching a consensus on certain decision points. However, with technical support provided by UNDP experts in terms of facilitating discussions, explaining the different functions of the health sector to stakeholders from other sectors and bridging the said gaps, they were able to overcome such a challenge. It may therefore be inferred that such forms of intermediation are important for the success of this exercise.

Participants of the taskforces were inclined to identify the decision points through process mapping. This proved to be useful to generate an exhaustive list of decision points at a detailed level. However, ultimately all decision points will have to be aggregated at the level of health system functions in order to be able to assess the ‘impact’ and therefore ‘risk’ of any given deviation. For that reason, it is recommended to look at both processes and functions when identifying decision points.

Assessments – that is, the assignment of values to impact and likelihood, particularly drivers and restraints – are more effective with a qualitative – not quantitative – scoring system. When the latter format was tried out, participants were observed focusing on assigning a number rather than having a collective snapshot that considers all the contextual variables. Moreover, the assessment of risk is far from an exact science; it requires judgment and is impacted by personal biases. Excessive focus on quantification of risk through a scoring system may backfire because, firstly, the scoring process varies among individuals: for example ‘very likely’ might be assigned a 2.5/5 by one person and a 4.5/5 by another even if they agree exactly on the nature of the likelihood. Moreover, some drivers and restraints are difficult to quantify or weigh. For example, it is difficult to quantitatively compare a very strong physical control and a very weak accounting system. These two restraints do not realistically balance each other out (even though numerically speaking, they might). Judgment and qualitative assessment maybe more effective as input to policy reform. Such assessments may be prone to bias, but these biases can be minimized by selecting diverse multi-stakeholder groups.

Despite the differences across the national contexts, some health sector issues are common and consistent across the Arab region. One significant commonality pertains to the priority health functions. The heat maps developed by the national joint task forces indicate that service delivery (especially hospital-level service delivery) and supply of medicine are shared national concerns with regard to corruption vulnerability. Of course, these are the priorities given the nature of today’s context and so they may change with the evolving dynamics related to Universal Health Care. However, irrespective of the details of these dynamics, adopting a similar approach to addressing corruption and documenting findings has created opportunities for knowledge and experience sharing and facilitated comparisons among countries. This in turn creates prospects for common reforms and agendas to arise on the regional level and for ensuing collective concerted action to address them.

### Development and implementation limitations

This research is the output of participatory observation whereby the development of the Conceptual Framework was conducted in conjunction with its implementation. This created constraints with respect to sampling, country context, and availability of information. The hypersensitivity of the topic and the nature of the setting further constrained access to individuals and information.

As for implementation challenges, one key issue is the fact that public frustration is better assuaged with corruption crackdowns where actual heads roll. Accordingly, there is generally greater demand for criminalization and enforcement; rather than prevention which, by default, avoids finger-pointing.

Another key challenge relates to the setup of the multi-stakeholder groups due to, as aforementioned, the prevalent lack of trust from all sides.

## Conclusion

Traditional governance reforms to health systems are unlikely to be effective in achieving the SDGs because they are typically undermined by corruption. The Conceptual Framework, as a tool for reform, addresses corruption as the core entry point; it focuses on specific decision points and transactions rather than broad governance issues. The Framework goes beyond the traditional approaches of sanctions and awareness raising and focuses on prevention through risk management. Tailored to the specificities of the health sector and the local context, this approach may be effective in driving concrete integrity efforts by means of creating a common language and agenda among different stakeholders, changing the mindset towards reform, and developing targeted solutions. As such, it may be capable of generating observable and sustainable progress towards healthcare reform and therefore of serving as an excellent platform for the Global Network on Anti-corruption, Transparency and Accountability in the Health Sector (GNACTA) to launch further research work-streams and country-level initiatives.

## Paper context

UNDP-ACIAC has been engaged in building the capacities of anti-corruption communities in Arab countries. With the aim to move from generic punitive approaches to preventative sector-specific approaches for combating corruption, a risk-based approach was developed and implemented and national capacities were built in several countries. This paper aims to document the conceptual development of the methodology and pilot implementation experiences of the preventative risk-based approach to corruption risk management in the health sector.
